# Temperature-Dependent Regulation of Co-Occurring Toxins, Odor Compounds, and Disinfection By-Product Precursors in Two Bloom-Forming Species

**DOI:** 10.3390/life15121933

**Published:** 2025-12-17

**Authors:** Lixia Shang, Yunyan Deng, Xiang Bai, Muhua Feng

**Affiliations:** 1 Laboratory of Marine Ecology and Environmental Sciences, Institute of Oceanology, Chinese Academy of Sciences, Qingdao 266071, China; yunyandeng@qdio.ac.cn; 2 State Key Laboratory of Lake Science and Environment, Nanjing Institute of Geography and Limnology, Chinese Academy of Sciences, Nanjing 210008, China; 3 Department of Pharmaceutical and Bioengineering, Zibo Polytechnic University, Zibo 255300, China; baix04@163.com

**Keywords:** microcystins, trihalomethanes, haloacetic acids, *β*-cyclocitral, *β*-ionone

## Abstract

Cyanobacterial blooms pose significant threats to aquatic ecosystems and drinking water safety, primarily through the release of diverse secondary metabolites. This study systematically explored the dynamics of secondary metabolites in *Microcystis aeruginosa* and *Anabaena* sp. under controlled conditions, focusing on the effects of temperature (10 °C, 25 °C, 35 °C) and growth phases (exponential, stationary, decline). Key parameters measured included cell density, dissolved organic carbon (DOC), microcystins (MC-LR, MC-RR), taste and odor compounds (*β*-cyclocitral, *β*-ionone), and disinfection by-product formation potentials (trihalomethanes (THMs) and haloacetic acids (HAAs)). Results revealed striking interspecific differences: *M. aeruginosa* exhibited significantly higher metabolite production, with peak DOC, extracellular MC-LR, and particulate *β*-cyclocitral observed in the decline phase at 25–35 °C. In contrast, *Anabaena* sp. showed an “early accumulation advantage” for THM precursors and “residual release” in the decline phase. Temperature played a critical regulatory role, with 25 °C as the optimal for most metabolites, while 35 °C enhanced extracellular release of dissolved *β*-cyclocitral in *M. aeruginosa*. Growth phase dynamics were consistent across species, with stationary and decline phases marked by elevated metabolite concentrations due to intensified synthesis and cell lysis, particularly for HAAs. These findings highlight species-specific metabolic strategies and their environmental drivers, providing critical insights for assessing and managing cyanobacterial bloom risks in aquatic ecosystems.

## 1. Introduction

In recent decades, the escalating frequency and intensity of cyanobacterial blooms in eutrophic freshwater ecosystems have emerged as a pressing global environmental concern, posing severe threats to aquatic ecological balance and drinking water security [1,2,3]. Driven by anthropogenic nutrient loading (e.g., excessive nitrogen and phosphorus inputs) and climate change-induced warming, blooms dominated by cyanobacteria such as *Microcystis aeruginosa* and *Anabaena* sp. have become ubiquitous in lakes, reservoirs, and rivers worldwide [4,5,6,7,8]. These blooms are not merely a visual nuisance; their ecological impacts extended far beyond biomass accumulation, primarily through the release of a diverse array of secondary metabolites [1,9]. These metabolites, including hepatotoxic microcystins (MCs), taste and odor (T/O) compounds, and precursors of disinfection by-products (DBPs), collectively contributed to a cascade of environmental and public health risks [10,11,12,13,14,15].

The ecological and health hazards associated with cyanobacterial metabolites were multifaceted and interconnected [12,16]. Microcystins, the most studied cyanotoxins, are cyclic heptapeptides produced predominantly by *Microcystis* spp., with MC-LR and MC-RR being the most common and toxic isoforms [17,18,19,20,21,22]. These toxins exerted potent inhibitory effects on protein phosphatases, leading to liver damage in aquatic organisms and humans upon prolonged exposure, with the World Health Organization (WHO) establishing a strict drinking water guideline of 1 μg·L^−1^ for MC-LR [23,24,25,26]. Concurrently, T/O compounds including *β*-cyclocitral and *β*-ionone, the degradation product of cyanobacterial carotenoids, impaired water palatability even at nanogram levels, causing significant economic losses for water utilities due to increased treatment costs and public complaints [27,28,29,30,31]. With the intensification of eutrophication, algae and their secretions (algogenic organic matter, AOM) in water bodies had become a crucial component of natural organic matter in many surface water systems [32,33,34,35]. Yet another aspect that had been gaining increased attention, despite being less recognized, was the equally critical role of cyanobacterial metabolites: they acted as precursors of DBPs, which were formed during chlorination—the most commonly employed drinking water disinfection method [36,37,38,39]. Trihalomethanes (THMs) and haloacetic acids (HAAs), the primary classes of DBPs, are known carcinogens and mutagenogens, with their formation potential being tightly linked to the composition and concentration of cyanobacteria-derived organic matter [40,41,42,43].

The production and release of these metabolites were tightly regulated by environmental factors, with growth phase and temperature emerging as important drivers [31,44]. Cyanobacteria underwent distinct physiological transitions across growth phases—lag, exponential, stationary, and decline—each associated with unique metabolic priorities [45]. During the exponential phase, resources were primarily allocated to cell division, resulting in minimal metabolite release; in contrast, the stationary phase, triggered by nutrient limitation, might have induced a shift toward secondary metabolism [45,46]. The decline phase, characterized by massive cell lysis, further exacerbates metabolite release as intracellular contents are passively discharged into the water column [47]. Cyanobacteria also exhibited a similar periodic growth process in natural waters, with their growth stages generally including the lag phase, development phase, maintenance phase, and decay phase [45,48]. Specifically, if cyanobacterial blooms occur in drinking water source areas, water intake at different stages of these blooms may exert distinct impacts on the selection of tap water treatment processes [31]. Cyanobacteria were shown to be favored in an increasingly warmer environment, with a positive relationship between elevated mean water temperatures and cyanobacterial biomass levels [49,50]. Optimal temperatures enhanced photosynthetic efficiency and carbon fixation, providing sufficient precursors for metabolite synthesis, while extreme temperatures could either suppress or disrupt metabolic pathways, leading to altered metabolite profiles [38,51].

Despite extensive research on cyanobacterial metabolites, critical knowledge gaps remained, particularly regarding interspecific differences and their implications for risk assessment. *Microcystis aeruginosa* and *Anabaena* sp. are two dominant bloom-forming cyanobacteria with distinct ecological strategies: *Microcystis* often forms large, toxic colonies, while *Anabaena* is capable of nitrogen fixation via specialized heterocysts [7,52]. However, comparative studies on their metabolite dynamics—including toxins, T/O compounds, and DBP precursors—across growth phases and temperature gradients are limited. Existing research has predominantly focused on single metabolite classes or individual species, failing to capture the complexity of multi-metabolite interactions and species-specific responses to environmental drivers [31,53,54]. For instance, while *Microcystis* is known for high MC production, the extent to which *Anabaena* contributes to DBP formation or T/O compound release, and how these contributions vary with growth stage and temperature, remains poorly understood. Such gaps hinder the development of targeted bloom management strategies and accurate risk predictions.

To address these limitations, this study systematically investigated the dynamics of key metabolites in *M. aeruginosa* and *Anabaena* sp. under controlled laboratory conditions. Specifically, we quantified dissolved organic carbon (DOC) release, MC concentrations (MC-LR and MC-RR), T/O compound levels (*β*-cyclocitral and *β*-ionone), and DBP formation potentials (THMs and HAAs) across three growth phases (exponential, stationary, decline) and three temperatures (10 °C, 25 °C, 35 °C). By comparing these two ecologically dominant species, we aim to: (1) characterize interspecific differences in metabolite production and release; (2) elucidate the combined effects of growth phase and temperature on metabolite dynamics; (3) identify key risk periods and conditions associated with elevated toxin, odor, and DBP precursor levels. The findings of this study are expected to enhance our understanding of cyanobacterial metabolite regulation, inform targeted bloom mitigation efforts, and improve drinking water treatment strategies for cyanobacteria-impacted sources. Ultimately, this work contributed to the broader goal of safeguarding freshwater ecosystems and public health in the face of escalating cyanobacterial bloom occurrences.

## 2. Materials and Methods

### 2.1. Laboratory Cultivation, Density and DOC Determination of Cyanobacteria

Monocultures of cyanobacteria were purchased from the Institute of Hydrobiology, Chinese Academy of Sciences (Wuhan, China), including *M. aeruginosa* (FACHB-912) and *Anabaena* sp. (FACHB-709). Due to the relatively high content of organic matter (citric acid, EDTA) in the standard BG11 medium, which interfered with the determination of algogenic organic matter and the formation of DBPs, a modified BG11 medium was used in relevant experiments [55]. The modified BG11 medium comprised two categories of components at the following final concentrations: (1) Major components: 1500 mg·L^−1^ NaNO_3_, 40 mg·L^−1^ K_2_HPO_4_·3H_2_O, 20 mg·L^−1^ Na_2_CO_3_, 13.6 mg·L^−1^ CaCl_2_, 75 mg·L^−1^ MgSO_4_·7H_2_O, 0.5 mg·L^−1^ FeCl_3_, 0.5 mg·L^−1^ Na_2_EDTA. (2) Trace components: 0.715 mg·L^−1^ H_3_BO_3_, 0.4525 mg·L^−1^ MnCl_2_·4H_2_O, 0.0555 mg·L^−1^ ZnSO_4_·7H_2_O, 0.01975 mg·L^−1^ CuSO_4_·5H_2_O, 0.0975 mg·L^−1^ Na_2_MoO_4_·2H_2_O [55]. After preparation, the pH of the medium was adjusted to 7.5 ± 0.1 using 1 M NaOH or HCl to ensure optimal growth conditions for the cyanobacteria [55]. Cyanobacteria were cultured using a sequencing batch method: 3 L of medium was added to 5 L white vials, sterilized, and cultured in batches. The light intensity was 2000 lux with a light:dark cycle of 12 h:12 h. The temperatures were set to 10 °C, 25 °C, and 35 °C, as they collectively cover the full spectrum of cyanobacterial bloom-related temperature conditions in freshwater systems (i.e., low-temperature pre-bloom initiation, moderate-temperature peak bloom optimization, and high-temperature extremality under climate change), aligned with field observation data (including our unpublished data of 32–38 °C in Lake Chaohu’s cyanobacterial bloom hotspots) and ecological characteristics of cyanobacteria documented in relevant studies [16,31]. During cultivation, the cultures were manually and gently inverted 2–3 times daily to prevent cell sedimentation at the flask bottom and ensure consistent contact between cyanobacterial cells and the growth medium. To investigate the growth characteristics of cyanobacteria, the algal cultures grown at each temperature were sampled every 3~7 days. Each sample was carefully but quickly taken from the middle of the culture bottle to ensure uniformity in the bottle. The densities were determined by counting the number of cells through microscopic observation (Nikon, TS100F, Nikon Instruments Inc., Tokyo, Japan) according to Brierley et al. [56]. At least 400 individual cells or filaments were counted to control the counting error below 15%. Algal solution at specific growth stages including exponential, stationary and decline phase was collected and centrifuged at 7000× *g* to separate the supernatant and algal cells. The supernatant was filtered through a GF/C membrane (Whatman, Cytiva, Marlborough, MA, USA) to measure DOC using a TOC analyzer (Shimadzu TOC 5000A, Shimadzu Corporation, Kyoto, Japan).

### 2.2. Determination of Microcystins

Three MCs variants including MC-LR, -RR and -YR with extracellular forms (EMC-LR, EMC-RR and EMC-YR) and intracellular forms (IMC-LR, IMC-RR and IMC-YR) were extracted and condensed from water samples using the method modified according to Barco et al. [57] and Shang et al. [16]. Samples were filtered through GF/C (Whatman) glass fiber membranes. The filtrate was used for the determination of extracellular MCs, and algal cells retained on the membrane were used for intracellular MCs. Total MCs concentration was the sum of extracellular and intracellular MCs. C18 solid-phase extraction (SPE) cartridges were activated with 10 mL methanol and 10 mL ultrapure water. Filtered water samples were passed through the cartridges at a flow rate of 8–10 mL·min^−1^. After enrichment, the cartridges were rinsed with 20% methanol solution to remove impurities, then eluted with 10 mL acidified methanol (methanol containing 0.1% trifluoroacetic acid), and the eluent was collected in glass tubes. The eluent was rotary-evaporated to near dryness at 40 °C, washed into glass tubes with 3–5 mL methanol, and dried under nitrogen. Finally, the residue was dissolved in 50% methanol aqueous solution to a volume of 100–500 μL, transferred to vials, stored at −20 °C, and determined by high-performance liquid chromatography. Membranes containing algal cells were repeatedly freeze-thawed three times at −20 °C and 4 °C, cut into pieces, and placed in 40 mL brown glass bottles. 15 mL of 5% acetic acid solution was added, shaken on a shaker for 30 min, and centrifuged to obtain supernatant. 20 mL of 75% methanol aqueous solution was added to the residue, disrupted using an ultrasonic cell disruptor under ice bath conditions (observed under a microscope until cells were completely broken), and centrifuged to obtain supernatant. Another 20 mL of 75% methanol aqueous solution was added to the residue, shaken on a shaker for 30 min, and centrifuged to obtain supernatant [57]. The three supernatants were combined, and subsequent enrichment and concentration steps were the same as those for extracellular MCs.

Microcystins were detected using a high-performance liquid chromatography (Agilent HPLC 1200, Agilent Technologies, Santa Clara, CA, USA) equipped with a diode array detector (DAD). The chromatographic column was an Agilent Eclipse XDB-C18 (5 μm, 4.6 × 150 mm) with a detection wavelength of 238 nm, column temperature of 25 °C, injection volume of 20 μL, and flow rate of 1 mL·min^−1^. Mobile phase A was aqueous solution containing 0.05% trifluoroacetic acid; mobile phase B was acetonitrile. The gradient program was: initial 70% A + 30% B, linear elution to 60% A + 40% B over 0–15 min, and equilibrated for 5 min. Qualitative analysis was based on the spectra and retention times of MC-RR, MC-YR, and MC-LR standards; quantitative analysis was based on peak areas. After concentrating the samples, the detection limits for the three MC congeners in intra- and extra-cellular forms were at the 0.01 µg·L^−1^ level.

### 2.3. Determination of Taste and Odor Compounds

The T/O compounds in samples were analyzed via solid-phase microextraction combined with gas chromatography-mass spectrometry (GC-MS) according to Watson et al. [58] and Shang et al. [16]. Samples were filtered through GF/C glass fiber membranes to separate the dissolved and particulate T/O compounds. Dissolved T/O compounds were extracted using headspace extraction. 80 mL of filtrate was accurately measured into a 125 mL headspace vial, added with 20 g of analytical-grade NaCl (baked at 550 °C for 2 h using the Ceramic Fiber Muffle Furnace (TM-0912P, Beijing Ying’an Meicheng Scientific Instrument Co., Ltd., Beijing, China)) and a magnetic stir bar, and sealed with a cap containing a polytetrafluoroethylene membrane. The extraction fiber was inserted through the cap, and extraction was performed under magnetic stirring in a 65 °C water bath for 30 min, followed by GC/MS determination. For the particulate T/O compounds, membranes containing algal cells were folded, repeatedly freeze-thawed three times at −20 °C and 4 °C, cut into pieces, placed in brown headspace vials, added with ultrapure water, and disrupted using an ultrasonic cell disruptor under ice bath conditions until no intact cells were detected; the volume was adjusted to 80 mL. Subsequent steps were the same as those for dissolved T/O compounds.

The T/O compound samples were stirred and extracted using a 2 cm long CAR/DVB/PDMS fiber (Supelco, Sigma-Aldrich Co. LLC, St. Louis, MO, USA) at 65 °C for 30 min before injection into the GC-MS. The T/O compounds, subsequent to extraction, underwent analysis utilizing an Agilent 6890 gas chromatograph interfaced with an Agilent 5973 mass spectrometer, equipped with a DB-5MS column (Agilent Technologies, Inc., Santa Clara, CA, USA). The GC temperature program involved an initial holding phase at 60 °C for 2 min, followed by a gradual increase to 200 °C (maintained at a constant temperature for 2 min) at a rate of 8 °C·min^−1^, and subsequently elevated to 260 °C at a rate of 15 °C·min^−1^. Data analysis was executed in the selected ion monitoring mode. The identification of the targeted T/O compounds was accomplished through the comparison of retention times with established standards, alongside the verification of characteristic ions at *m*/*z* 112 and 126 for geosmin, 107 and 95 for 2-methyl isoborneol, 137 and 152 for *β*-cyclocitral, and *m*/*z* 177 and 91 for *β*-ionone. Quantification of each analyte was carried out using the corresponding standard curve, with detection limits established at 0.2, 0.2, 0.5, and 0.4 ng·L^−1^ for geosmin, 2-methyl isoborneol, *β*-cyclocitral, and *β*-ionone, respectively.

### 2.4. Formation and Determination of Disinfection By-Products

For formation potential experiment, reactions were conducted in 100 mL sealed brown bottles with polytetrafluoroethylene (PTFE) caps. Specifically, total DBPs here referred to data obtained from chlorination experiments conducted directly on raw algal culture (including algal cells and extracellular organic matter (EOM)); in contrast, extracellular DBPs denoted data derived from chlorination experiments on extracellular organic matter, which was isolated by filtering the raw algal culture through a GF/C filter to remove algal cells. Since no bromide ions were present in the culture medium, only the formation potential of chlorinated products, including chloroform (TCM), monochloroacetic acid (MCAA), dichloroacetic acid (DCAA) and trichloroacetic acid (TCAA), were determined. A certain volume of algal sample was added, free chlorine was added according to the organic carbon concentration (5 mg·L^−1^), and the reaction pH was controlled at 7.5 using 10 mM phosphate buffer. The initial chlorine concentration was quantified by DPD/FAS titration. After 7 days of reaction in the dark at 25 °C, residual chlorine concentration was measured (between 3 and 5 mg·L^−1^), and the reaction was terminated with ascorbic acid or sodium sulfite. Residual chlorine (free chlorine and total chlorine) was determined using the DPD/FAS titration method [59].

According to the United States Environmental Protection Agency (USEPA) Method 551.1, liquid–liquid extraction of THMs from samples was performed using methyl tert-butyl ether (MTBE), followed by detection with gas chromatography coupled with an electron capture detector (GC/ECD) [60]. For THM determination, 3.0 mL of MTBE and 10 g of baked anhydrous sodium sulfate were added to the sample, and the mixture was vortexed until the sodium sulfate was completely dissolved. Next, the mixture was allowed to stand for 30 min to ensure complete phase separation. Once separated, the upper organic layer was transferred to a GC vial using a disposable pipette, with care taken to ensure no water remained at the bottom of the vial—if two phases were present, the bottom water layer was removed using a Pasteur pipette, and the remaining MTBE phase was transferred to another clean vial. The resulting extracts were stored below 10 °C and analyzed by GC/ECD within 14 days, where samples were qualitatively identified based on retention time and quantified via the external standard method [55]. HAAs in samples were extracted using MTBE, derivatized with methanol, and detected by GC/ECD [61]. In detail, 1 mL of concentrated sulfuric acid was added to the water sample, followed by 8 g of baked anhydrous sodium sulfate, after which the cap was closed and the mixture was vortexed until the sodium sulfate was completely dissolved. Next, 4 mL of MTBE containing an internal standard was added, the mixture was subjected to vortex extraction for 3 min, and then left to stand for 30 min to achieve complete phase separation. Subsequently, 2 mL of the upper organic layer was transferred to a 10 mL screw-cap test tube with a PTFE cap, 2 mL of acidified methanol (a methanol solution containing 10% sulfuric acid) was added, and the contents were mixed well. The test tube was then placed in a 50 ± 2 °C water bath for a 2 h reaction; after cooling, 5 mL of a 150 g·L^−1^ sodium sulfate solution was added, the mixture was shaken, and left to stand for phase separation. Using a disposable pipette, the lower aqueous layer was removed, leaving no more than 0.3 mL of aqueous solution. After that, 1 mL of saturated sodium bicarbonate solution was added in four portions—after each addition, the mixture was mixed, the cap was opened to release gas, and the test tube was left to stand for 5 min. Finally, the upper organic layer was transferred to a GC vial, the extracts were stored below 10 °C and analyzed by GC/ECD within 14 days, with samples qualitatively identified by retention time and quantified by the internal standard method [55]. The instrumental parameters for the GC analysis were set as follows: the injector temperature was maintained at 210 °C; the temperature of the ECD was set to 320 °C; and the GC oven temperature program was configured as follows: an initial temperature of 38 °C was held for 20 min, followed by ramping up to 100 °C at a heating rate of 10 °C per minute with a subsequent 5 min hold at this temperature, and then further ramping up to 250 °C at a heating rate of 25 °C per minute, which was held for 2 min. The total run time for the entire analysis process was 39.2 min.

### 2.5. Data Processing

All experiments were performed in triplicate to ensure reproducibility. Graphs were plotted using Origin 9.0 to visualize trends in metabolite concentrations across growth phases and temperatures. IBM SPSS Statistics 27 software was used to perform one-way ANOVA to compare differences in metabolite concentrations among temperature groups or growth phases, followed by Tukey’s HSD post hoc test for pairwise comparisons when the ANOVA result was significant. Statistical significance was defined as *p* < 0.05, and highly significant differences were denoted as *p* < 0.01.

## 3. Results

### 3.1. Variation in Growth Curve and DOC Concentration

**Figure 1:**
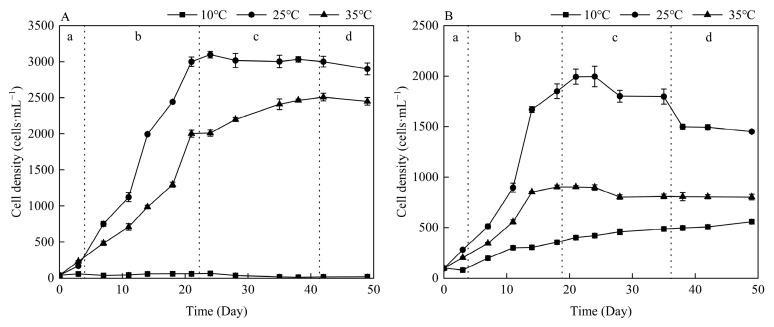
Growth curves of *M. aeruginosa* (**A**) and *Anabaena* sp. (**B**) under different temperature conditions. a, lag phase; b, exponential phase; c, stationary phase; d, decline phase.

Different temperature cultivation conditions exerted distinct influences on the growth trajectories of *M. aeruginosa* and *Anabaena* sp. (Figure 1). Both species achieved peak growth at 25 °C, yet their tolerances to extreme temperatures diverged significantly. At 10 °C, *M. aeruginosa* remained in a stagnant state with minimal cell density increase, while *Anabaena* sp. exhibited slow but continuous growth—suggesting *Anabaena* sp. possessed greater cold resistance. Conversely, at 35 °C, *M. aeruginosa* maintained approximately 80% of its maximum cell density observed at 25 °C (3.1 × 10^7^ cells·mL^−1^), whereas *Anabaena* sp. only reached about 50% of its 25 °C-derived peak (2.0 × 10^7^ cells·mL^−1^).

**Figure 2:**
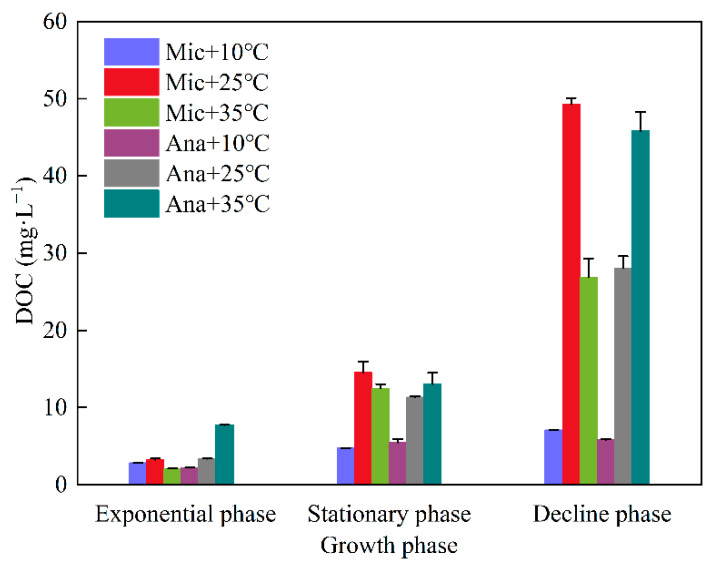
DOC variation in *M. aeruginosa* (Mic) and *Anabaena* sp. (Ana) across growth phases at different temperatures (10 °C, 25 °C, 35 °C).

Dissolved organic carbon concentrations of *M. aeruginosa* and *Anabaena* sp. under different temperatures and growth phases were analyzed (Figure 2). Overall, DOC levels exhibited a marked upward trend across growth phases. In the exponential phase, DOC was lowest (mostly <10.0 mg·L^−1^) for all groups, indicating minimal DOC release during initial growth. During the stationary phase, DOC increased (typically 10.0–20.0 mg·L^−1^ at 25 °C and 35 °C) as cyanobacterial metabolism and lysis intensified. The decline phase saw peak DOC (some > 40.0 mg·L^−1^) due to massive cell lysis. Species-wise, for *M. aeruginosa*, the DOC levels during the three growth phases at 25 °C were higher than those at 10 °C and 35 °C. The concentration reached its peak at 25 °C (49.3 mg·L^−1^) during the decline phase, which had 1.8-fold higher DOC than *Anabaena* sp. at 25 °C (28.1 mg·L^−1^). In contrast, for *Anabaena* sp., the DOC levels followed the order of 35 °C > 25 °C > 10 °C. Moreover, the DOC levels at 35 °C throughout the three growth phases were consistently higher than those of *M. aeruginosa*.

### 3.2. Microcystin Concentration Variations in Microcystis aeruginosa and Anabaena sp.

**Figure 3:**
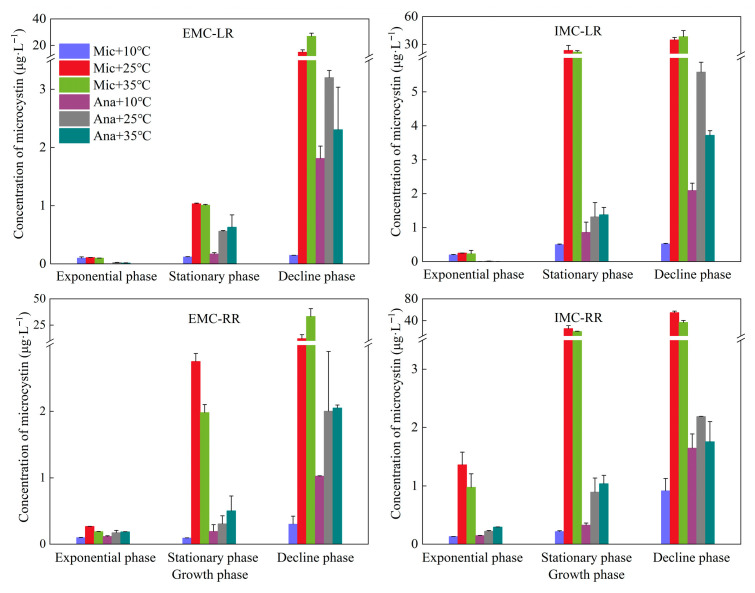
Variations in microcystin concentrations under different temperature conditions at different growth phases. Mic, *M. aeruginosa*; Ana, *Anabaena* sp. E, extracellular concentration; I, intracellular concentration.

Analysis of MC concentration variations in *M. aeruginosa* and *Anabaena* sp. across different growth phases and temperatures revealed distinct patterns (Figure 3). The MC isoforms produced by both algal species were identified as MC-LR and MC-RR, while MC-YR was not detected in all the samples. Extracellular and intracellular MC concentrations in both cyanobacterial species increased consistently as cultures progressed from exponential to stationary to decline phases. This trend reflects the enhanced cellular lysis, metabolism, and subsequent toxin release associated with cyanobacterial senescence. In the logarithmic phase, except for the concentration of IMC-RR from *M. aeruginosa* (up to 1.4 μg·L^−1^), the concentrations of all other detected substances (EMC-LR, IMC-LR and EMC-RR) were less than 0.3 μg·L^−1^. In decline phase, the concentrations of both intracellular and extracellular MCs produced by *Anabaena* sp. ranged between 1.0 and 2.2 μg·L^−1^ at 10 °C, which were significantly higher than the corresponding yields of M. aeruginosa (less than 0.9 μg·L^−1^, *p* < 0.05). However, the *M. aeruginosa* demonstrated significantly greater MC production and release capacity than *Anabaena* sp. at 25 °C and 35 °C, with the disparity being especially pronounced during the decline phase (*p* < 0.05). Specifically, during the stationary phase, the MCs content of *M. aeruginosa* was higher at 25 °C than at 35 °C, while *Anabaena* sp. showed the exact opposite trend. In the decline phase, among the MCs produced by *M. aeruginosa*, except for IMC-RR, the concentrations at 25 °C were all lower than those at 35 °C. The maximum concentrations of EMC-LR, IMC-LR, EMC-RR, and IMC-RR were 27.0, 38.5, 33.4, and 55.0 μg·L^−1^, respectively. For *Anabaena* sp., except for EMC-RR, the concentrations of MCs at 25 °C were all higher than those at 35 °C. The maximum concentrations of EMC-LR, IMC-LR, EMC-RR, and IMC-RR were 3.2, 5.6, 2.0, and 2.2 μg·L^−1^, respectively.

**Figure 4:**
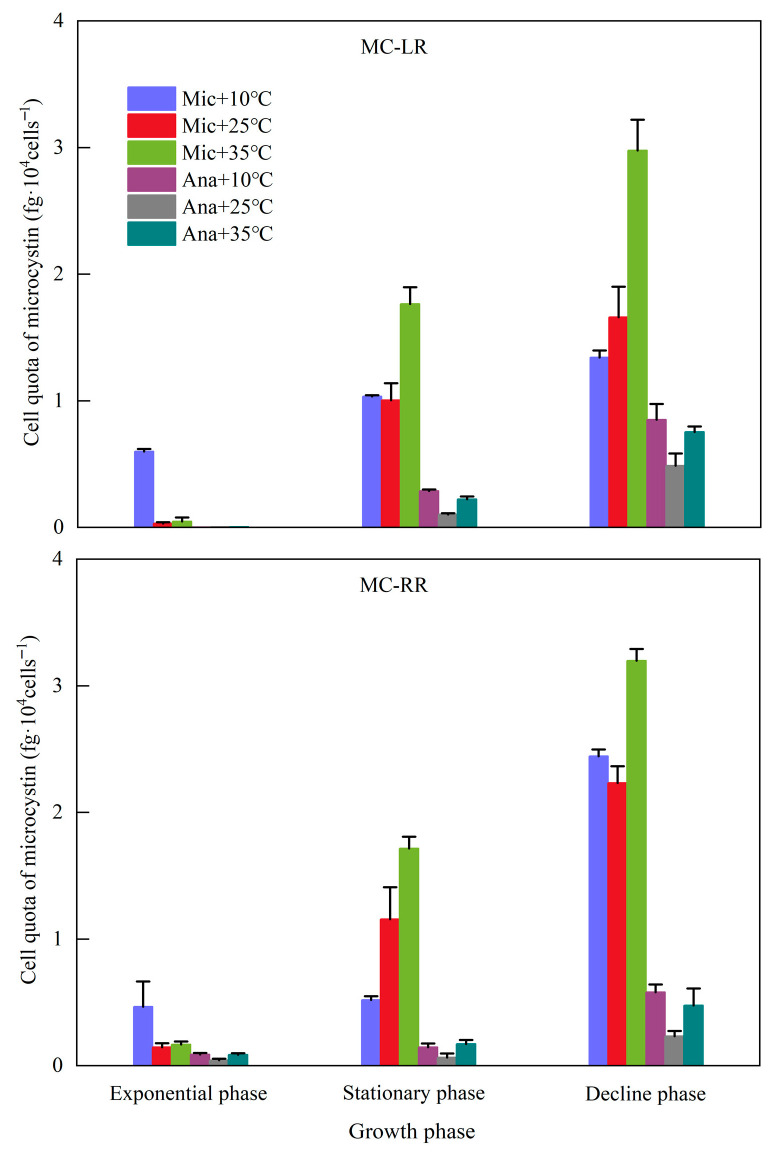
Variations in cell quotas of microcystins under different temperature conditions at different growth phases. Mic, *M. aeruginosa*; Ana, *Anabaena* sp.

The dynamics of MC cell quotas, including MC-LR and MC-RR, in *M. aeruginosa* and *Anabaena* sp. were systematically analyzed across different growth phases and temperature regime (Figure 4). The MC cell quotas of both cyanobacterial species exhibited a distinct increasing trend with the progression of growth phases. For *M. aeruginosa*, during the exponential phase, due to the low cell density, the MC cell quotas at 10 °C were higher than those at 25 °C and 35 °C. Across the three growth phases, the MC cell quotas at 35 °C were all higher than those at 25 °C; the concentrations of MC-LR and MC-RR reached 3.0 and 3.2, respectively, in the decline phase. For *Anabaena* sp., the MC cell quotas were consistently lower than those of *M. aeruginosa*. Moreover, except for the MC-RR in the stationary phase, the MC cell quotas followed the order of 10 °C > 35 °C > 25 °C.

### 3.3. Dynamics of T/O Compounds in Microcystis aeruginosa and Anabaena sp.

**Figure 5:**
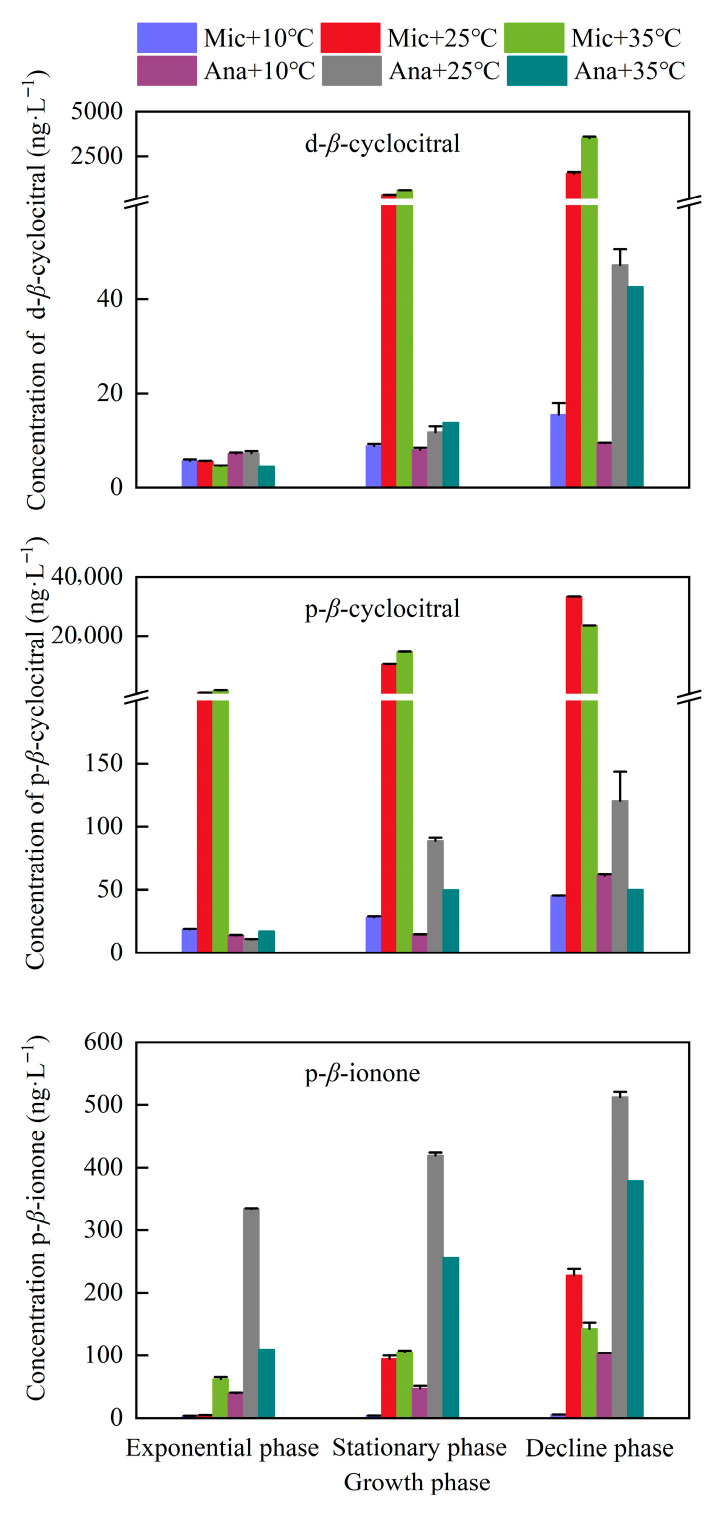
Variations in T/O compounds under different temperature conditions at different growth phases. Mic, *M. aeruginosa*; Ana, *Anabaena* sp. d, dissolved compounds; p, particulate compounds.

To elucidate the dynamics of secondary metabolites in cyanobacteria, we systematically analyzed the concentration variations of dissolved and particulate T/O compounds in *M. aeruginosa* and *Anabaena* sp. across different growth phases and temperature regimes (Figure 5). Two T/O compounds *β*-cyclocitral and *β*-ionone were detected. Among them, *β*-ionone was exclusively identified in the intracellular fraction, while a large proportion of *β*-cyclocitral was also present within the cells. In the exponential growth phase, the concentration of dissolved *β*-cyclocitral produced by *M. aeruginosa* was consistently below 5.7 ng·L^−1^. However, in the stationary phase, the concentration of dissolved *β*-cyclocitral increased rapidly under the cultivation conditions of 25 °C and 35 °C. This increasing trend continued into the decline phase, with the maximum concentration (3529.3 ng·L^−1^) observed at 35 °C. Regarding the particulate *β*-cyclocitral produced by *M. aeruginosa* in the exponential phase: a relatively low concentration (less than 18.5 ng·L^−1^) was detected under the 10 °C cultivation condition; in contrast, under the cultivation conditions of 25 °C and 35 °C, the concentrations reached 784.4 ng·L^−1^ and 1494.6 ng·L^−1^, respectively. Subsequently, the concentration of particulate *β*-cyclocitral continued to increase in the stationary phase and decline phase, peaking in the decline phase at 33,457.6 ng·L^−1^ (for 25 °C) and 23,540.2 ng·L^−1^ (for 35 °C). The concentration of *β*-cyclocitral produced by *Anabaena* sp. was significantly lower than that produced by *M. aeruginosa* (*p* < 0.05). Nevertheless, it also increased with the extension of cultivation time. The maximum concentrations of dissolved and particulate *β*-cyclocitral in *Anabaena* sp. were 42.7 ng·L^−1^ and 120.5 ng·L^−1^, respectively, which were detected in the decline phase under the 25 °C cultivation condition. As for *β*-ionone, the concentration of particulate *β*-ionone produced by *Anabaena* sp. was higher than that produced by *M. aeruginosa* under all cultivation conditions. Both *Anabaena* sp. and *M. aeruginosa* exhibited the maximum concentration of *β*-ionone in the decline phase under the 25 °C cultivation condition, with the values being 419.7 ng·L^−1^ and 227.6 ng·L^−1^, respectively.

### 3.4. Dynamics of Formation Potential of DBPs for Microcystis aeruginosa and Anabaena sp.

**Figure 6:**
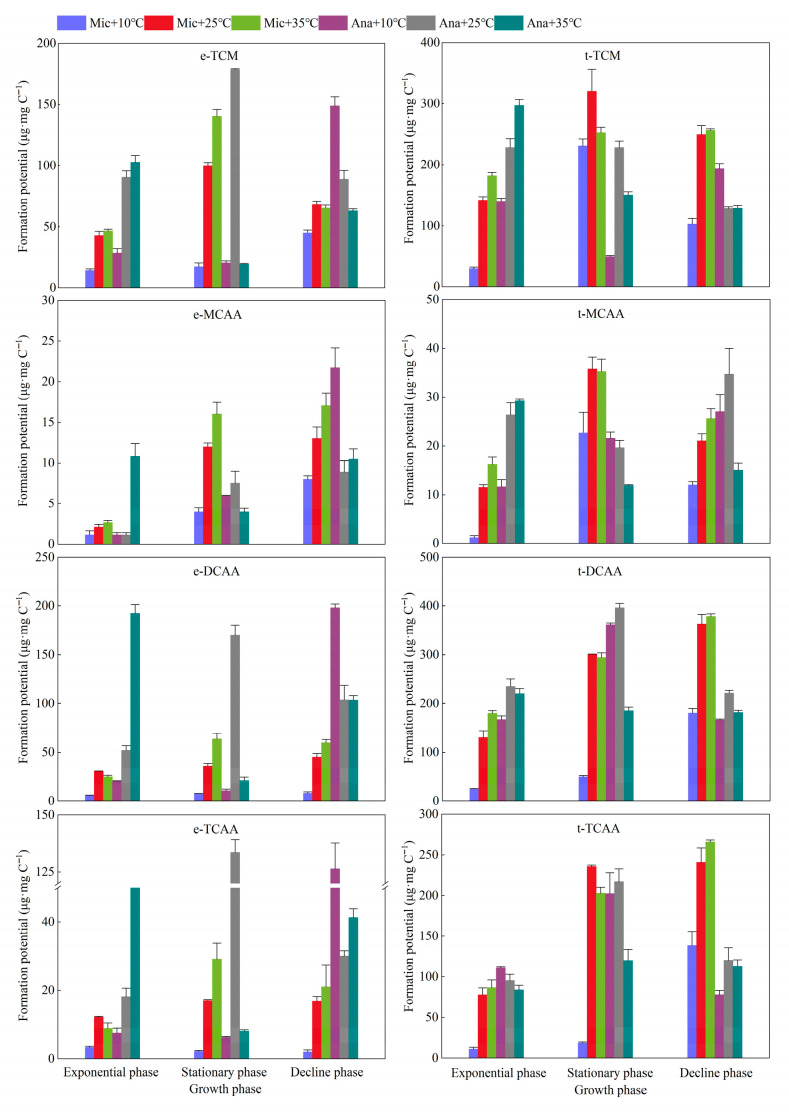
Variations in the trihalomethane and haloacetic acids formation potential of cyanobacteria and organic matter under different temperature conditions at different growth phases. Mic, *M. aeruginosa*; Ana, *Anabaena* sp.; e, extracellular organic matter (EOM); t, total organic matter including algal cells and extracellular organic matter.

The dynamic variations in the formation potential of TCM in *M. aeruginosa* and *Anabaena* sp. were investigated across different growth phases and temperature regimes (Figure 6). For *M. aeruginosa*, the formation potential ranges of extracellular TCM and total TCM were 14.1–140.4 μg·mg C^−1^ and 29.6–320.6 μg·mg C^−1^, respectively. The TCM formation potential at 10 °C was significantly lower than that at 25 °C and 35 °C (*p* < 0.01). At 25 °C and 35 °C, there was no significant difference in the formation potential of extracellular TCM between the exponential and decline phases, while the highest yield of extracellular TCM was observed in the stationary phase. Notably, the yield of extracellular TCM at 35 °C (140.4 μg·mg C^−1^) was significantly higher than that at 25 °C (100.0 μg·mg C^−1^)(*p* < 0.05). For the formation potential of total TCM, the maximum values at 10 °C and 25 °C were both achieved in the stationary phase, reaching 230.9 μg·mg C^−1^ and 320.6 μg·mg C^−1^, respectively. At 35 °C, the formation potential of total TCM increased from 181.9 μg·mg C^−1^ in the exponential phase to 252.3 μg·mg C^−1^ in the stationary phase and further to 256.6 μg·mg C^−1^ in the decline phase. For *Anabaena* sp., the formation potential ranges of extracellular TCM and total TCM were 49.6–297.2 μg·mg C^−1^ and 19.5–178.6 μg·mg C^−1^, respectively. Across all three tested temperatures, the maximum TCM formation potential occurred in the stationary phase, and the lowest yield per unit carbon was detected at 10 °C. Additionally, the TCM formation potential of *Anabaena* sp. (from both EOM and raw algal culture) increased with rising temperature in the exponential phase; the highest yield was observed at 25 °C in the stationary phase, whereas the highest yield in the decline phase was recorded at 10 °C. At 10 °C, the TCM formation potential followed the order: decline phase > exponential phase > stationary phase. At 25 °C, the formation potential of extracellular TCM peaked in the stationary phase at 178.6 μg·mg C^−1^, while the formation potential of total TCM remained consistent at 228.1 μg·mg C^−1^ in both the exponential and stationary phases. At 35 °C, both the formation potential of extracellular TCM and that of total TCM reached their maxima in the exponential phase, with values of 102.8 μg·mg C^−1^ and 297.2 μg·mg C^−1^, respectively.

For *M. aeruginosa*, the extracellular MCAA increased with temperature and growth stage, reaching a maximum of 17.0 μg·mg C^−1^ at 35 °C and in the decline phase (Figure 6). The total MCAA content followed the order of stationary phase > decline phase > exponential phase, and reached a maximum of 35.8 μg·mg C^−1^ at 25 °C. For *Anabaena* sp., at 10 °C and 25 °C, the extracellular MCAA increased with the growth stage, reaching a maximum of 21.7 μg·mg C^−1^ at 10 °C and in the decline phase. At 35 °C, there was no significant difference in extracellular MCAA between the exponential and decline phases, with a value of approximately 10 μg·mg C^−1^, which was higher than that in the stationary phase. For the total MCAA, it increased with the growth cycle at 10 °C. At 25 °C, the total MCAA in the decline phase was the highest (34.7 μg·mg C^−1^), followed by the exponential phase and then the stationary phase. At 35 °C, the total MCAA in the exponential phase was the highest (29.2 μg·mg C^−1^), and the order was exponential phase > decline phase > stationary phase.

Both extracellular DCAA and total DCAA for *M. aeruginosa* increased with the growth stage, reaching their maxima in the decline phase. Regarding extracellular DCAA, except that its content at 25 °C was slightly higher than that at 35 °C in the exponential phase, the content in the stationary and decline phases followed the order of 35 °C > 25 °C > 10 °C, with the maximum value of 63.8 μg·mg C^−1^ observed in the stationary phase. As for total DCAA, in the exponential and decline phases, its content followed the order of 35 °C > 25 °C > 10 °C; however, in the stationary phase, the content at 25 °C was slightly higher than that at 35 °C. The maximum value of total DCAA, 378.5 μg·mg C^−1^, was achieved at 35 °C in the decline phase. For *Anabaena* sp., the extracellular DCAA reached its highest content at 35 °C, 25 °C, and 10 °C in the exponential, stationary, and decline phases, respectively, with the corresponding values being 198.2 μg·mg C^−1^, 169.9 μg·mg C^−1^, and 192.5 μg·mg C^−1^. For total DCAA, the highest content was observed at 25 °C across all three growth phases, and the maximum value of 396.3 μg·mg C^−1^ was attained in the stationary phase.

The extracellular TCAA for *M. aeruginosa* reached its highest content at 25 °C in the exponential phase. In the stationary and decline phases, however, the extracellular TCAA content increased with rising temperature, peaking at 29.1 μg·mg C^−1^ at 35 °C. As for the total TCAA, its content generally increased with the progression of the growth stage. The highest total TCAA content was observed at 25 °C in the stationary phase. In the exponential and decline phases, the total TCAA content increased with increasing temperature, and the maximum value of 265.8 μg·mg C^−1^ was achieved in the decline phase. For *Anabaena* sp., the extracellular TCAA content in the exponential phase increased with rising temperature. In the stationary phase, it reached the highest value of 133.7 μg·mg C^−1^ at 25 °C, while in the decline phase, the extracellular TCAA content was 126.4 μg·mg C^−1^ at 10 °C. Regarding the total TCAA, its content in the exponential phase slightly decreased with increasing temperature. The total TCAA content peaked in the stationary phase, reaching 217.0 μg·mg C^−1^ at 25 °C, and then declined in the decline phase. For *Anabaena* sp., the extracellular TCAA content in the exponential phase increased with rising temperature. In the stationary phase, it reached the highest value of 133.7 μg·mg C^−1^ at 25 °C, while in the decline phase, the extracellular TCAA content was 126.4 μg·mg C^−1^ at 10 °C. Regarding the total TCAA, its content in the exponential phase slightly decreased with increasing temperature. The total TCAA content peaked in the stationary phase, reaching 217.0 μg·mg C^−1^ at 25 °C, and then declined in the decline phase.

## 4. Discussion

The frequent occurrence of cyanobacterial blooms in eutrophic waters has become a critical environmental issue threatening freshwater ecosystems and drinking water safety globally [1,2,3,4]. This study focused on *M. aeruginosa* and *Anabaena* sp., systematically investigating the dynamics of growth, DOC release, MCs synthesis, T/O compound production, and DBPs formation potential under different temperatures (10 °C, 25 °C, 35 °C) and growth phases (exponential phase, stationary phase, decline phase). We summarized the key results Table 1. The results revealed interspecific specificity, temperature dependence, and phase dynamics of cyanobacterial metabolites, providing key insights for understanding the ecological risks of cyanobacterial blooms and formulating targeted prevention strategies.

### 4.1. Regulatory Mechanisms of Metabolites: Synergistic Effects of Growth Phase, Temperature, and Interspecific Specificity

The growth phase of cyanobacteria was a core factor regulating metabolite dynamics, with both *M. aeruginosa* and *Anabaena* sp. exhibiting significant phase-dependent characteristics. During the exponential phase, both species prioritized biomass accumulation, resulting in minimal metabolite release, consistent with the physiological strategy of allocating carbon sources and energy primarily to cell division and proliferation [45,46]. Entering the stationary phase, intensified nutrient competition and increased cell density triggered metabolic reprogramming, activating secondary metabolic pathways: *M. aeruginosa* showed significant increases in MCs (MC-LR, MC-RR) and *β*-cyclocitral concentrations, while *Anabaena* sp. exhibited advantages in accumulating TCM precursors. During the decline phase, massive cell lysis led to extensive release of intracellular metabolites, with DOC, MC and *β*-cyclocitral concentrations reaching maximum values, consistent with the physiological process of passive release of intracellular substances after cyanobacterial cell structure disintegration [47]. This phase-dependent difference stemmed from the temporal regulation of gene expression. The toxin synthesis gene clusters in *M. aeruginosa* were activated by nitrogen regulatory factors during the stationary phase, while the nitrogen-fixing genes in *Anabaena* sp. were expressed during the exponential phase, leading to early accumulation of TCM precursors [53,62,63]. Additionally, oxidative stress in stationary-phase cells (e.g., reactive oxygen species from photosystems) could induce MCs synthesis, which acted as an antioxidant to protect cells from damage, explaining the significant increase in MC cell quota of *M. aeruginosa* during the stationary phase [64].

Temperature significantly regulated the production and release efficiency of cyanobacterial metabolites by affecting enzyme activity and cell membrane permeability [49,51,65]. In this study, 25 °C was confirmed as the optimal growth temperature for both *M. aeruginosa* and *Anabaena* sp.—both species achieved their peak cell densities at this temperature. Interspecific differences in temperature adaptability were particularly significant. *M. aeruginosa* could maintain approximately 80% of its maximum cell density at 35 °C, with extracellular MC-RR concentration reaching 33.4 μg·L^−1^. Its heat resistance may stem from high expression of heat shock proteins, which stabilized the structure of toxin synthesis-related enzymes [66,67]. In contrast, *Anabaena* sp. growth was inhibited at 35 °C, but DOC release during the decline phase exceeded that at 25 °C, possibly due to accelerated cell lysis at high temperatures, leading to passive release of intracellular organic matter (IOM). At low temperature (10 °C), the slow growth and continuous metabolite release (e.g., TCM precursors) of *Anabaena* sp. reflected its adaptation to low-temperature environments, possibly related to the expression of cold shock proteins [68,69]. However, 25 °C did not always serve as the golden temperature for metabolite synthesis, as evidenced by the distinct temperature preferences of the two species for producing key metabolites. For *M. aeruginosa*, several critical metabolites showed higher yields at 35 °C rather than 25 °C. For instance, the decline phase—a stage marked by massive cell lysis and metabolite release—saw higher concentrations of extracellular MCs and total MCs (except IMC-RR) at 35 °C than at 25 °C; the maximum MCs concentrations far exceeding those at 25 °C. Similarly, dissolved *β*-cyclocitral peaked at 35 °C in the decline phase, and extracellular TCM formation potential at 35 °C was higher than that at 25 °C in the stationary phase. For HAAs, extracellular MCAA of *M. aeruginosa* reached its maximum at 35 °C in the decline phase, and total DCAA also peaked at 35 °C in the decline phase. In contrast, *Anabaena* sp. exhibited higher metabolite production at 10 °C or 35 °C depending on the metabolite type. The MC cell quotas of *Anabaena* sp. at 10 °C or 35 °C were significantly higher than that of 25 °C during the stationary and decline phases (*p* < 0.05). The extracellular DCAA and MCAA reached the maximum at 35 °C in the exponential phase and at 10 °C in the decline phase, respectively. This discrepancy—where the optimal growth temperature does not align with the optimal metabolite synthesis temperature—might be attributed to the distinct physiological demands of growth versus secondary metabolism. At 25 °C, both cyanobacterial species likely exhibit the highest photosynthetic efficiency and carbon fixation rate, which might prioritize carbon allocation to cell proliferation rather than secondary metabolite production [65,70]. In contrast, non-optimal growth temperatures (10 °C or 35 °C) may induce stress responses: for example, 35 °C accelerates cell lysis and metabolic turnover in *M. aeruginosa*, promoting the release of pre-synthesized metabolites and activating pathways for toxin and T/O compound production; 10 °C, while inhibiting rapid growth of *Anabaena* sp., may redirect carbon flux toward synthesizing stress-related secondary metabolites to enhance survival. Thus, 25 °C provided ideal conditions for biomass accumulation but might fail to supply sufficient carbon precursors or trigger the stress signals required for efficient secondary metabolite synthesis.

The metabolic differences between *M. aeruginosa* and *Anabaena* sp. essentially resulted from long-term niche adaptation. *M. aeruginosa* adopted a “high-investment, high-risk” strategy: it inhibited competitors by synthesizing high concentrations of MCs and *β*-cyclocitral and resisted grazing through thick-walled colony structures [53,71,72]. The expanded non-ribosomal peptide synthetase in its genome might provide a molecular basis for this strategy [73]. *Anabaena* sp. adopted a “resource allocation trade-off” strategy: it allocated more energy to nitrogen fixation (heterocyst differentiation), resulting in lower secondary metabolite production (e.g., maximum MC-LR of 3.2 μg·L^−1^), but occupied a temporal niche in competition through the “early accumulation + residual release” dynamics of TCM precursors [62,63].

### 4.2. Ecological Risk Assessment: Combined Hazards of Metabolites and Challenges to Drinking Water Safety

The high toxicity of *M. aeruginosa* poses significant threats to aquatic ecosystems. The MC-LR released during its decline phase far exceeded the WHO drinking water limit (1 μg·L^−1^), which can cause liver damage in animals and reduce fecundity in aquatic invertebrates by inhibiting protein phosphatases [74,75]. Research data show that the MC cell quota of *M. aeruginosa* reached 3.0 fg·cell^−1^ during the decline phase at 35 °C; based on its cell density, a single population can produce a toxin load sufficient to harm the entire food web [76]. Meanwhile, high concentrations of *β*-cyclocitral could not only cause water odor but also inhibited phytoplankton diversity by damaging algal cell membranes, forming an ecological trap of “single-species dominance” [77,78]. This allelopathic effect was particularly significant during the stationary phase, with the maximum particulate *β*-cyclocitral concentration of *M. aeruginosa* being 277-fold that of *Anabaena* sp., which might contribute to its dominance in cyanobacterial community succession [11].

The ecological risks of *Anabaena* sp., although characterized by low toxicity, are covert and long-term. The DOC released during its decline phase at 35 °C reached 45.8 mg·L^−1^, which is rich in polysaccharides and amino acids and can serve as a carbon source for heterotrophic bacteria, accelerating the eutrophication cycle of water bodies [79,80]. In addition, the TCM precursors accumulated by *Anabaena* sp. during the decline phase at 10 °C were not easily degraded in low-temperature water bodies and could maintain DBPs formation potential for a long time, becoming a hidden danger in drinking water treatment during winter [16,31,81]. The metabolites of the two cyanobacteria formed combined pollution of “toxins-odor-DOC” in water bodies, exerting cascading effects on ecosystem structure and function: toxins inhibit zooplankton grazing, odor compounds reduce water palatability, and high DOC exacerbates dissolved oxygen consumption, leading to the death of organisms [11,47,82].

This study systematically revealed the differences in risks between the two cyanobacteria as precursors of DBPs, providing key data for drinking water safety assessment. *Microcystis aeruginosa* displayed an overall pattern of “high-temperature driven, concentrated outbreak during the stationary to decline phases” for HAAs formation: high temperatures (25 °C and 35 °C) significantly enhanced the synthesis and release efficiency of HAAs precursors (such as amino acids and proteins). The concentrations of most HAAs increased continuously with the progression of growth phases, reaching peaks during the decline phase due to cell lysis. This formed a risk pattern characterized by “precursor accumulation in the stationary phase and explosive release in the decline phase”. In contrast, *Anabaena* sp. demonstrated the characteristics of “broad temperature adaptability and scattered risk phases”: it broke the conventional understanding of “higher temperature leading to higher risk”. The HAAs risk was prominent during the decline phase at low temperature (10 °C); the stationary phase at medium temperature (25 °C) served as the core risk window for all HAAs; and the HAAs risk was relatively high during the exponential phase at high temperature (35 °C). Additionally, the overall concentration of total HAAs was the highest at 25 °C, which reflected a metabolic strategy of adapting to different temperatures by adjusting the timing of precursor synthesis and release. Therefore, the synergistic regulation of temperature and growth phase was the core factor driving HAAs formation in both species. For *M. aeruginosa*, temperature dominated the intensity of HAAs risk—high temperatures significantly raised the upper limit of risk—while the growth phase determined the timing of risk, with the stationary and decline phases constituting the key risk window. For *Anabaena* sp., temperature mainly affected the growth phase at which HAAs reach their peak, causing the risk window to shift with temperature changes; the growth phase, on the other hand, determined the degree of risk contribution under different temperatures, ensuring that *Anabaena* sp. maintained the ability to accumulate HAAs precursors in diverse environments.

The risks of cyanobacterial metabolites showed a progressive “low-high-extremely high” trend with growth phases, and the dominant risks varied across phases. The exponential phase was characterized by the early accumulation of TCM and HAA precursors in *Anabaena* sp.; although the overall risk was low at this stage, it laid the groundwork for subsequent DBP formation. The stationary phase was the outbreak period of toxins and odor substances in *M. aeruginosa*, posing direct threats to aquatic organisms and the sensory quality of drinking water. The decline phase, due to massive cell lysis, saw peak concentrations of all metabolites, forming superimposed pollution of “toxins-odor-DBP precursors”, which was the stage with the highest ecological risks and water treatment pressure [12,16,83].

Furthermore, the interspecific difference in phase risks also provided precise targets for bloom management: *M. aeruginosa*-dominated blooms require focused monitoring from the stationary to the decline phase, while *Anabaena* sp. blooms need early intervention during the exponential phase. In addition, the coexistence of the two algae may lead to risk superimposition; for example, the combination of high toxins from *M. aeruginosa* and high TCM precursors from *Anabaena* sp. during high-temperature summer periods would significantly increase the difficulty of drinking water treatment.

Therefore, conventional water treatment processes have significant bottlenecks in removing cyanobacterial metabolites, and the results of this study revealed the underlying mechanisms. Coagulation-sedimentation could remove some algal cells, but shear forces might cause the release of IOM from *M. aeruginosa*, which could directly increase DBP formation risks [84]. Chlorination could degrade some MCs, but might react with *β*-cyclocitral to generate more odorous oxidation products and promote the synthesis of nitrogenous DBPs (e.g., dichloroacetonitrile), which was closely related to the high organic nitrogen content (e.g., amino acids) in the IOM of *M. aeruginosa* [85,86,87].

### 4.3. Management Strategies and Prospects: Precision Prevention Based on Metabolic Laws

Based on the metabolic patterns of the two cyanobacterial species mentioned above, precision prevention and control should be developed around a three-dimensional framework of “species specificity-growth phase-temperature response” to achieve targeted management of the risks posed by cyanobacterial metabolites. For *M. aeruginosa*, the high-risk period of its metabolites (toxins, T/O compounds, HAA precursors) concentrated on the stationary to decline phases, with 25–35 °C being the window for high metabolite production. Therefore, intervention should be initiated in the late exponential growth phase, while enhanced coagulation should be implemented to remove biomass before massive cell lysis occurs, thereby reducing the subsequent release of metabolites [88]. For *Anabaena* sp., the “early accumulation” and “residual release during the decline phase” of its THM precursors were prominent characteristics, and it exhibits strong adaptability to low temperatures. Thus, focus should be placed on the exponential growth phase in low-temperature seasons (e.g., around 10 °C). Biological manipulation can be used to inhibit its early growth, and during the decline phase, the interception of dissolved organic matter should be strengthened to reduce the formation potential of DBPs.

From a technical perspective, existing drinking water treatment processes need to be optimized according to the metabolic differences between the two cyanobacteria: when treating water dominated by *M. aeruginosa*, a combined process of “pre-oxidation-enhanced coagulation-ozone-biological activated carbon” can be adopted, with a focus on degrading high concentrations of MCs and *β*-cyclocitral produced from the stationary to decline phases [89]. For water dominated by *Anabaena* sp., the pretreatment stage under low-temperature conditions should be strengthened, such as adding potassium permanganate for pre-oxidation to reduce the input of trihalomethane precursors [90].

In addition, a combined early warning system of “metabolites-environmental factors” needs to be established: for the period dominated by *M. aeruginosa*, MC-LR and total dichloroacetic acid should be used as core indicators; for the period dominated by *Anabaena* sp., trihalomethane precursors should be the key monitoring targets. The control thresholds should be dynamically adjusted based on temperature and growth phases. In the future, it will also be necessary to integrate molecular ecology and water treatment engineering technologies to identify key regulatory genes in cyanobacterial metabolism, develop environmentally friendly agents that target and inhibit metabolite synthesis, and optimize process parameters to adapt to the metabolic characteristics of different cyanobacteria. Ultimately, this will enable a shift from “passive emergency response” to “proactive precision prevention and control”, ensuring the safety of freshwater ecosystems and the quality of drinking water supply.

## 5. Conclusions

This study revealed interspecific differences in metabolite dynamics between *M. aeruginosa* and *Anabaena* sp.: *M. aeruginosa* produced higher yields of toxins, odor substances, and DBP precursors at high temperatures, while *Anabaena* sp. was characterized by low-temperature adaptability and early TCM accumulation. These differences resulted from the synergistic effects of growth phases, temperature, and genomic adaptability, forming the niche differentiation strategies of the two cyanobacteria. The findings provide a new perspective for understanding the combined risks of cyanobacterial blooms and lay a scientific foundation for formulating “phase-targeted, interspecific-differentiated” management strategies. Future interdisciplinary research (e.g., molecular ecology, water treatment engineering) is expected to achieve precise control of cyanobacterial metabolites, ensuring the safety of freshwater ecosystems and drinking water supply.

## Figures and Tables

**Table 1:** Summary table of key results for *Microcystis aeruginosa* and *Anabaena* sp. across growth phases and temperatures.

Parameter	Species	Optimal Temperature for Production/Release	Key Growth Phase Dynamics	Notable Interspecific Differences
Cell density	*M. aeruginosa*	25 °C (peak: 3.1 × 10^7^ cells·mL^−1^); 35 °C retains ~80% of peak	Lag → Exponential (rapid growth) → Stationary (plateau) → Decline (lysis)	Higher heat tolerance (maintains growth at 35 °C); negligible growth at 10 °C
	*Anabaena* sp.	25 °C (peak: 2.0 × 10^7^ cells·mL^−1^); 35 °C retains ~50% of peak	Slow but continuous growth at 10 °C; growth inhibited at 35 °C	Higher cold tolerance (grows at 10 °C); lower heat tolerance than *M. aeruginosa*
Dissolved organic carbon (DOC)	*M. aeruginosa*	25 °C (peak: 49.3 mg·L^−1^ in decline phase)	Exponential (low: <10 mg·L^−1^) → Stationary (10–20 mg·L^−1^) → Decline (peak: >40 mg·L^−1^)	Higher DOC production overall; peak at 25 °C (decline phase)
	*Anabaena* sp.	35 °C (consistently higher than 25 °C/10 °C)	Similar phase trend; DOC at 35 °C exceeds *M. aeruginosa* across all phases	Lower peak DOC (28.1 mg·L^−1^ at 25 °C, decline phase); enhanced release at 35 °C due to cell lysis
Microcystins (MCs)	*M. aeruginosa*	35 °C (extracellular MCs); 25 °C (intracellular MCs except IMC-RR)	Exponential (low: <0.3 μg·L^−1^) → Stationary → Decline (peak: EMC-LR = 27.0 μg·L^−1^; IMC-RR = 55.0 μg·L^−1^)	Dominant MC producer; MC cell quota increases with growth phase; higher yields at 25–35 °C
	*Anabaena* sp.	10 °C (stationary/decline phases); 25 °C (IMC-LR/IMC-RR)	Exponential (low) → Stationary → Decline (peak: EMC-LR = 3.2 μg·L^−1^; IMC-LR = 5.6 μg·L^−1^)	Low MC production; higher MC cell quota at 10 °C/35 °C than 25 °C; MC concentrations < 5% of *M. aeruginosa*
Taste & odor (T/O) compounds	*M. aeruginosa*	35 °C (dissolved *β*-cyclocitral); 25 °C (particulate *β*-cyclocitral)	Exponential (low) → Stationary → Decline (peak: dissolved *β*-cyclocitral = 3529.3 ng·L^−1^; particulate *β*-cyclocitral = 33,457.6 ng·L^−1^)	Dominant *β*-cyclocitral producer; *β*-ionone peak at 25 °C (decline phase: 227.6 ng·L^−1^)
	*Anabaena* sp.	25 °C (both *β*-cyclocitral and *β*-ionone)	Gradual increase with growth phase; peak *β*-cyclocitral (dissolved = 42.7 ng·L^−1^; particulate = 120.5 ng·L^−1^)	Higher *β*-ionone production (peak: 419.7 ng·L^−1^ at 25 °C, decline phase); low *β*-cyclocitral yields
Trihalomethanes (THMs) formation potential	*M. aeruginosa*	25 °C (stationary phase: total TCM = 320.6 μg·mg C^−1^); 35 °C (extracellular TCM = 140.4 μg·mg C^−1^)	Exponential (low) → Stationary (peak) → Decline (sustained high)	Higher total THM potential at 25–35 °C; extracellular THM peak in stationary phase
	*Anabaena* sp.	25 °C (stationary phase: total TCM = 228.1 μg·mg C^−1^); 35 °C (exponential phase: extracellular TCM = 102.8 μg·mg C^−1^)	“Early accumulation” (exponential phase) → Stationary (peak) → Decline (high at 10 °C)	Lower total THM potential but earlier accumulation; THM risk scattered across phases/temperatures
Haloacetic acids (HAAs) formation potential	*M. aeruginosa*	35 °C (MCAA/DCAA); 25 °C (TCAA)	Exponential (low) → Stationary → Decline (peak: total DCAA = 378.5 μg·mg C^−1^; total TCAA = 265.8 μg·mg C^−1^)	HAAs increase with growth phase; high-temperature (25–35 °C) dominance; peak in decline phase
	*Anabaena* sp.	25 °C (stationary phase: total DCAA = 396.3 μg·mg C^−1^; total TCAA = 217.0 μg·mg C^−1^); 10 °C (MCAA)	Broad temperature adaptability; MCAA peak at 10 °C (decline phase: 21.7 μg·mg C^−1^); DCAA peak at 35 °C (exponential phase: 198.2 μg·mg C^−1^)	HAAs risk scattered across phases/temperatures; higher MCAA at low temperatures; peak TCAA at 25 °C

Note: Abbreviations: MC-LR (microcystin-LR), MC-RR (microcystin-RR), EMC (extracellular microcystin), IMC (intracellular microcystin), TCM (trichloroform), MCAA (monochloroacetic acid), DCAA (dichloroacetic acid), TCAA (trichloroacetic acid). Values represent maximum concentrations/formation potentials for each parameter across tested conditions. “Optimal Temperature” refers to the temperature yielding the highest value for the parameter (considering biological relevance to growth and metabolite synthesis).

## Data Availability

The datasets generated during and analyzed during the current study are available from the corresponding author on reasonable request.

## Abbreviations

The following abbreviations are used in this manuscript:

MCs	Microcystins
T/O	Taste and odor
DBPs	Disinfection by-products
DOC	Dissolved organic carbon
MC-LR	Microcystin-LR
MC-RR	Microcystin-RR
MC-YR	Microcystin-YR
EMC-LR	Extracellular microcystin-LR
IMC-LR	Intracellular microcystin-LR
EMC-RR	Extracellular microcystin-RR
IMC-RR	Intracellular microcystin-RR
EMC-YR	Extracellular microcystin-YR
IMC-YR	Intracellular microcystin-YR
THMs	Trihalomethanes
HAAs	Haloacetic acids
TCM	Chloroform
MCAA	Monochloroacetic acid
DCAA	Dichloroacetic acid
TCAA	Trichloroacetic acid
SPE	Solid-phase extraction
PTFE	polytetrafluoroethylene
GC-MS	Gas chromatography-mass spectrometry
HPLC	High-performance liquid chromatography
DAD	Diode array detector
GC/ECD	Gas chromatography-electron capture detector
MTBE	Methyl tert-butyl ether
AOM	Algogenic organic matter
IOM	Intracellular organic matter
EOM	Extracellular organic matter
WHO	World Health Organization
USEPA	United States Environmental Protection Agency
